# Cervical spine injury: clinical and medico-legal overview

**DOI:** 10.1007/s11547-022-01578-2

**Published:** 2023-01-31

**Authors:** Christian Zanza, Gilda Tornatore, Cristina Naturale, Yaroslava Longhitano, Angela Saviano, Andrea Piccioni, Aniello Maiese, Michela Ferrara, Gianpietro Volonnino, Giuseppe Bertozzi, Roberta Grassi, Fabrizio Donati, Michele Ahmed Antonio Karaboue

**Affiliations:** 1 Foundation “Ospedale Alba E Bra Onlus”, Verduno, Italy; 2 Research Training Innovation Infrastructure, Research and Innovation Department, Azienda Ospedaliera SS Antonio E Biagio E Cesare Arrigo, Alessandria, Italy; 3 Department of Emergency Medicine, Policlinico Gemelli/IRCCS- Catholic University of Sacred Heart, Rome, Italy; 4 Department of Anesthesia and Intensive Care Medicine, University of Milano Bicocca, Milan, Italy; 5 Emergency Medicine Residency Program, University of Pavia, Pavia, Italy; 6 Department of Emergency Medicine, Anesthesia and Critical Care Medicine, Michele and Pietro Ferrero Hospital, Verduno, Italy; 7 Department of Anesthesia and Critical Care Medicine-Critical Care Medicine Division, St. Antonio and Biagio Hospital, Alessandria, Italy; 8 Department of Surgical, Medical, and Molecular Pathology and Critical Care Medicine, Institute of Legal Medicine, University of Pisa, Pisa, Italy; 9 Department of Anatomical, Histological, Forensic and Orthopaedic Sciences, Sapienza University of Rome, Rome, Italy; 10 Department of Clinical and Experimental Medicine, Section of Legal Medicine, University of Foggia, Foggia, Italy; 11 Meyer Children’s Hospital, University of Florence, Florence, Italy; 12 Department of General Surgery, Orthopedic Institute, Bambino Gesù Children Hospital, Rome, Italy

**Keywords:** Cervical spine injury, NEXUS criteria, Canadian C-spine rule, Cervical spine imaging, Post-mortem imaging

## Abstract

Spinal trauma is an important cause of disability worldwide. Injury to the cervical spine (CS) occurs frequently after major trauma. 5–10% of patients with blunt trauma have a cervical spine injury. The cervical spine accounts for ~ 50% of all spinal injuries. Determination of CS stability is a common challenge in the acute care setting of patients with trauma. Several issues, indeed, are of particular concern: who needs CS imaging; what imaging should be obtained; when should computed tomography (CT), magnetic resonance imaging (MRI), or flexion/extension (F/E) radiographs be obtained; and how is significant ligamentous injury excluded in the comatose patient. CT and MRI both have roles to play. This article aims to present the different imaging to frame techniques to be used with greater precision in the acute event also for the purpose of planning the next therapeutic process. An overview of the applicability of the same methods in forensic pathology is also provided highlighting possible future biomarker to ease in diagnosis of acute TBI.

## Introduction

Spinal trauma is described as a notable cause of morbidity and mortality among young adults after road and workplace trauma worldwide, and it represents a significant proportion of musculoskeletal injuries from traumatic accidents [1]. In the USA, 150,000 people are affected annually, of which 11,000 suffer spinal cord damage. Cervical Spine (CS) injuries frequently occur within major trauma [2], among which 5–10% of patients have this lesion [3]. Moreover, CS accounts for ~ 50% of injuries affecting the whole spine. In the context of trauma, in the world, acute injuries of the cervical spine represent from 1.9 to 4.6 of subjects and up to 5.9% in the context of polytrauma [1].

Young men (M: F 4: 1), aged between 16 and 30 years, are the most affected by traumatic accidents, while the second peak of incidence concerns over 65-year-old people, leaving only 1–3% of events in people before age 15 [4]. Concerning the mechanisms of injury, they differ in different age groups: young men are usually involved in high-energy traumas, first and foremost road accidents, followed by traumas caused by falls, attacks, or sports, whereas in the over-65 group low-energy traumas are usually implicated [4]. In all the affected people, the mechanism of injury that leads to CS damage is consequent to the instability of the fractures of the bone component of CS. The most affected segment is the caudal part represented by C_6_ and C_7_, while in a third of cases C_2_ is affected. The spine, and specifically the cervical spine, is a particularly complex structure that has different components, each of which has its own characteristics of susceptibility to trauma and the ability to heal. Being a complex structure, there are countless varieties of lesions that can be found thus hindering the creation and comparison of homogeneous groups, classification, any therapeutic indications, and the effectiveness of different treatments. About that, early diagnosis remains extremely important to try to prevent or limit CS injuries, especially in cases of unstable fractures.

The most commonly used radiological examination in the study of CS trauma is most commonly based on the use of computed tomography even if the gold standard of the study of the spine is magnetic resonance [5].

Thus, this narrative review involved 47 articles was analyzed over 654 papers identified on PubMed and Google Scholar electronic databases, to present the imaging dockyard that have to be used with greater precision in the acute event also for the purpose of planning the next therapeutic approach. An overview of the applicability of the same diagnostic techniques in forensic field is also provided highlighting possible application of forensic radiology to ease in diagnosis of acute TBI.

### The classification proposed over time

As previously mentioned, spinal trauma involves a highly complex structure that has components with varying susceptibility both in terms of injury and healing potential.

In addition, the extremely complex anatomical and functional specifications make it difficult to create a classification of spinal injuries that can also generate specific therapeutic indications. In trauma to the spine, specifically the cervical one, the variables involved are neurological injuries, type and severity of bone component fractures, and its alignment with its degree of instability. A reliable classification system should consider all variables, be able to stratify lesions according to their severity, provide truthful information on prognosis, and guide clinical decision making. Finally, in order to be clinically useful, a classification must also be easy to apply, with good reproducibility, and must be understandable across the board between various specialists involved, using a common language. Even today, despite all efforts to generate it, a gold-standard classification system does not exist.

The first who created a classification system for spinal injuries, including the cervical compartment, was Holdsworth, in 1970 [6].

His classification system was based on a concept of stability. The stability derives from the integrity and correct functional integration between two anatomical–functional compartments of the vertebral column: the anterior column which guarantees the work in compression and the posterior column which acts as a tension band, and it is precisely the latter that, according to Holdsworth, it has the main role in the genesis and maintenance of the stability of the spine [6].

In 1983, Denis proposed a slightly different anatomical–functional model; he divided the column into anterior, central, and posterior. According to Denis, the compromise of the posterior column alone is not capable of generating clinically relevant instability by itself. In order to have instability, at least two out of three behaviors must be involved [7].

Allen and Ferguson proposed a different mechanistic classification, using conventional radiology and the dynamics of trauma. They thought of it divided the mechanism of injury into six categories: compressive flexion, distractive flexion, compressive extension, distractive extension, vertical compression, and lateral flexion. These were further subdivided according to the severity of the damage itself [8].

Over time, however, the mechanistic systems have not proved reliable enough, especially since radiological examinations clarify the mechanism of injury and do not take into account the energy that generates the trauma [9, 10].

The subsequent classifications have been conceived and built around objective elements of gravity.

In 2007, Vaccaro’s study group proposed the Subaxial Injury Classification (SLIC) and Severity Scale. The SLIC system takes into account the morphology, the neurological impairment, and the disco-ligamentous complex (DLC) integrity (Table 1) [9]. The disco-ligamentous complex is composed by intervertebral disc, facet capsules, and ligaments. It can be intact, indeterminate, displayed in magnetic resonance imaging as an insulated widening of the spinous process, or disrupted.

**Table 1:** Different classification system together with the treatment choice for each score

*SLIC system*			
Morphology	No abnormality Compression Burst Distraction Translation/Rotation	0 1 2 3 4	1 to 3 points: non-surgical management; 4 points: surgical or non-surgical management based on patient condition and surgeon preferences; 5 or more points: surgical management (realignment, stabilization ± decompression)[10–13]
DLC	Intact Indeterminate Disrupted	0 1 2	
Neurological status	Intact Nerve root injury Complete cord injury Incomplete cord injury Continuous cord compression + neurological deficit	0 1 2 3 + 1	
*Canadian C-spine rule (GCS 15)*			
Age > 65 years		1	< 1: no radiographic evaluation ≥ 1: cervical-spine radiography
Dangerous mechanism		1	
Paraesthesias in extremities		1	
No safe assessment of range of motion		1	
Unable to rotate the neck (45° left and right)		1	
*NEXUS Criteria (GCS 15)*			
Posterior midline cervical-spine tenderness		1	< 1: no radiographic evaluation ≥ 1: cervical-spine radiography
Evidence of intoxication		1	
Normal level of alertness		1	
Focal neurologic deficit		1	
Painful distracting injuries		1	

*Congress of neurological surgeons recommendation*	
Awake and asymptomatic not complaining of neck pain or tenderness, with a normal neurological examination and a complete functional range of motion at a physical examination	Radiographic evaluation of the cervical spine is not recommended and discontinuance of cervical immobilization is possible (Level I recommendation) [26]
Awake and symptomatic	High-quality computed tomography (CT) imaging is recommended If high-quality CT imaging is not available, 3 CS projections (anteroposterior, lateral, and odontoid) are recommended. Moreover, if necessary to defineor better visualize suspicious areas, a CT is recommended, as soon as available (Level I recommendation) [26]
Obtunded or unevaluable	High-quality CT imaging is recommended as the initial imaging technique of choice. If high-quality CT imaging is not available, 3 CS projections (anteroposterior, lateral, and odontoid) are recommended. Moreover, if necessary to define or better visualize suspicious areas, a CT is recommended, as soon as available (Level I recommendation) [26]

The system works assigning a rating to each of these elements, and the total score will indicate the recommended treatment [10–13].

According to the final score, the recommended treatment is the following:

- 1 to 3 points: non-surgical management;
- 4 points: surgical or non-surgical management based on patient condition and surgeon preferences;
- 5 or more points: surgical management (realignment, stabilization ± decompression) [10–13].

In 2008, two AO Spine Knowledge Forum Trauma groups revised the literature and proposed AOMagerl [14]. In this classification, we recognize the three classic injury models: compression injury (Type A), anterior and posterior distraction injury (Type B), dislocation / translation injury (Type C).

This system also allows for the inclusion in the evaluation of particular conditions that can guide the clinician in his decision-making process. These elements are defined as follows: M1 posterior capsule–ligamentous concussion injury, M2 trauma disc herniation, M3 spinal stiffening, and M4 vertebral artery signs [15].

AOMagerl also evaluates the possible state of joint damage by dividing them into the following options: F1 fracture of a single facet joint (lesion < 1 cm and < 40% of the area), F2 larger fractures (lesions > 1 cm and > 40%) up to floating lateral masses F3 or subluxated F4.

The degree of impairment of the neurological status is also considered in this classification system. It is identified with N0 when the patient is neurologically free, N1 had a transient neurological deficit that has completely resolved at the time of the clinical examination, N2 incomplete radiculopathy, N3 incomplete radiculopathy, up to complete lesions of the spinal cord identified with N4. The NX subtype describes an indeterminate neurological state [15].

In summary, there are multiple systems to grade and classify spinal traumatic injuries. Each of these classification is based on different elements and evaluates various aspects of the spine injuries. The McAfee and Denis scales evaluate the spinal instability of thoracolumbar injuries, and they are based on a 2 or 3 columns concept, whereas the Magerl classification takes into account the mechanism of injury based on CT findings [16].

### Mechanisms of injury

**Fig. 1:**
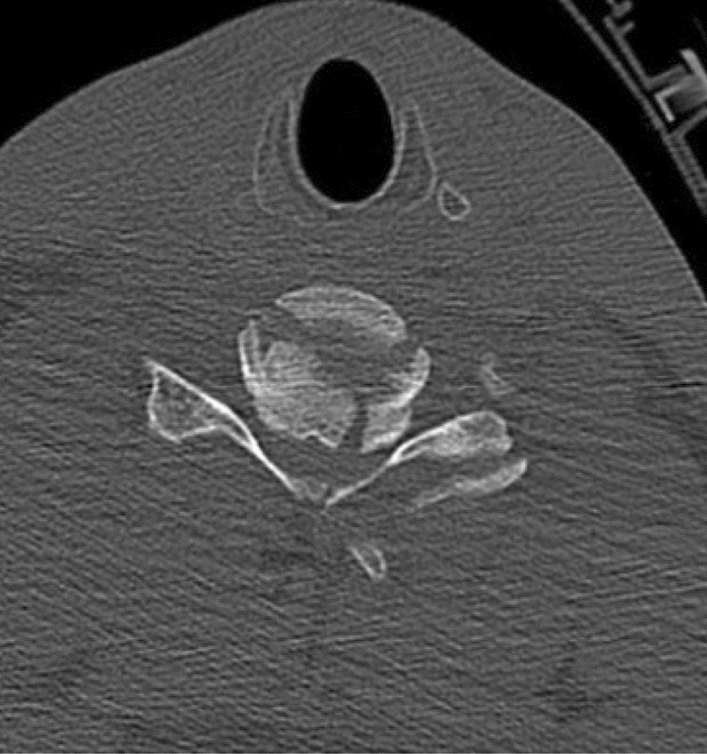
Burst fracture, CT scan axial view

**Fig. 2:**
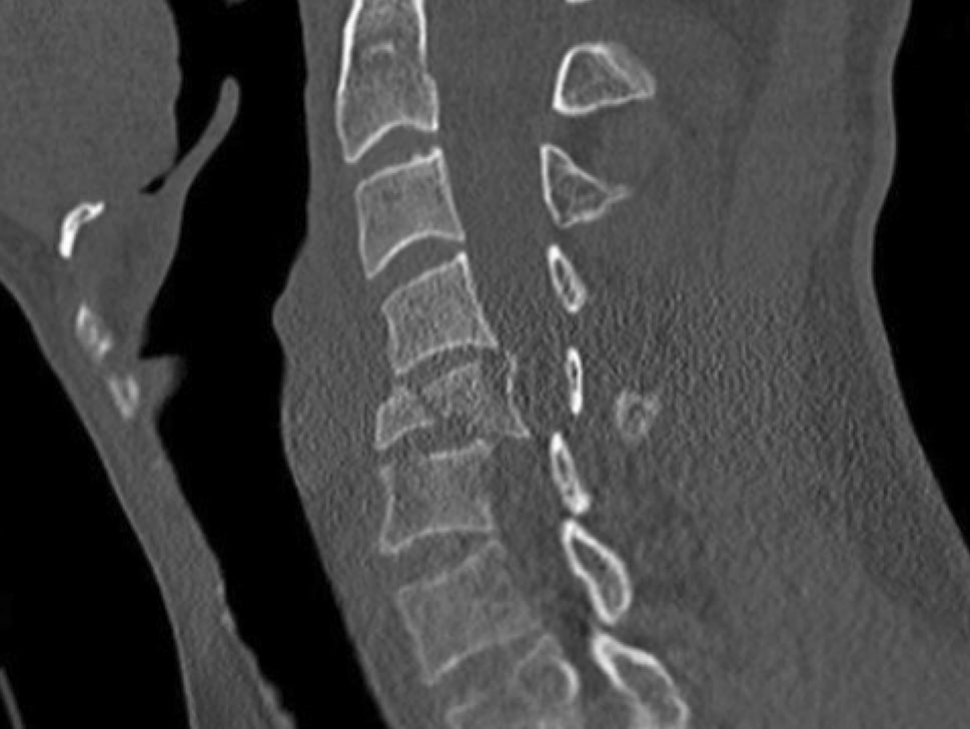
Burst fracture, CT scan, sagittal view

There are four main mechanisms of spinal injury, depending on the major forces applied during trauma. Each of them produces a recognizable radiologic pattern, or “footprint” [17–20]:

- *Axial compression mechanism (burst fracture)* (Figs. 1, 2);

Burst fractures are usually associated with high energy trauma and characterized by a loss of somatic height. Fracture instability is determined by element posteriorly displacemeed and/or vertebral body or facet dislocation or subluxation [18, 20] (figures 1,2).

**Fig. 3:**
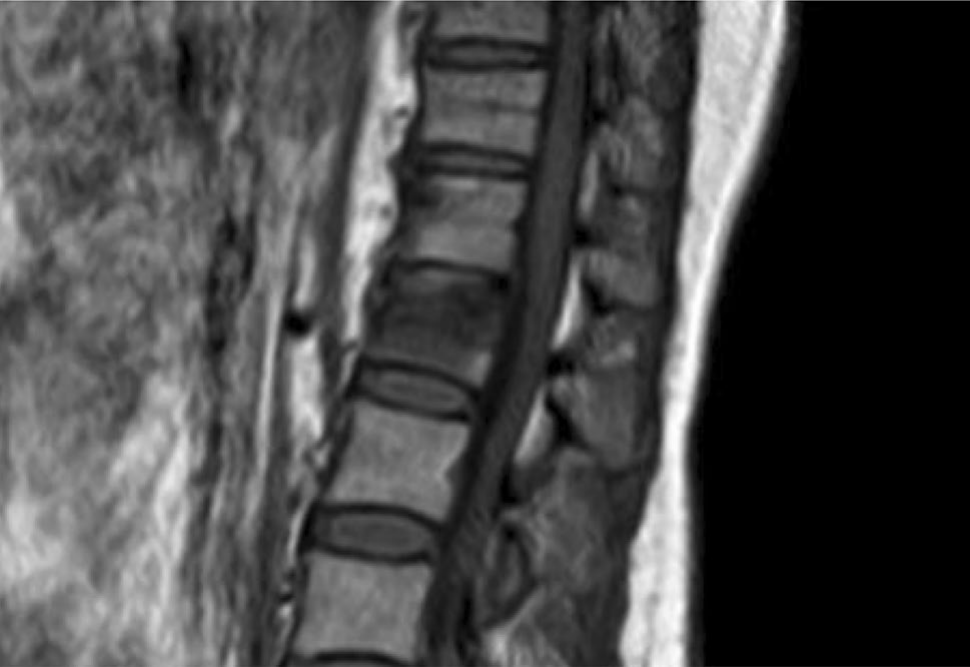
Wedge fracture*, MRI, sagittal view*

- *Flexion compression mechanism (wedge or compression fracture)* (Fig. 3)

This mechanism usually causes an anterior wedge compression fracture, with a compression of the anterior columns and variable involvement of the middle and posterior column [18].

- *Distraction mechanism*

It causes an anatomical dislocation of the MSs along the spine sagittal axis; it is usually associated with high energy forces, strong enough to overcome the strength of ligamentous system of suppot. This type of injury predisposes to fracture instability [20].

The association of flexion and distraction forces can cause a Chance-type fracture. The most common type of Chance fracture is characterized by a transversal fracture and a disruption of the supraspinous ligament, highlighted by the “dissolving pedicle sign,’’ characterized by a progressive loss of definition of pedicle on CT scans [20].

The association of extension and distraction forces, on the other hand, causes the disruption of the anterior disc, the anterior disc space is enlarged, with or without posterior column fracture, and the vertebral body can be displaced anteriorly or posteriorly [20].

**Fig. 4:**
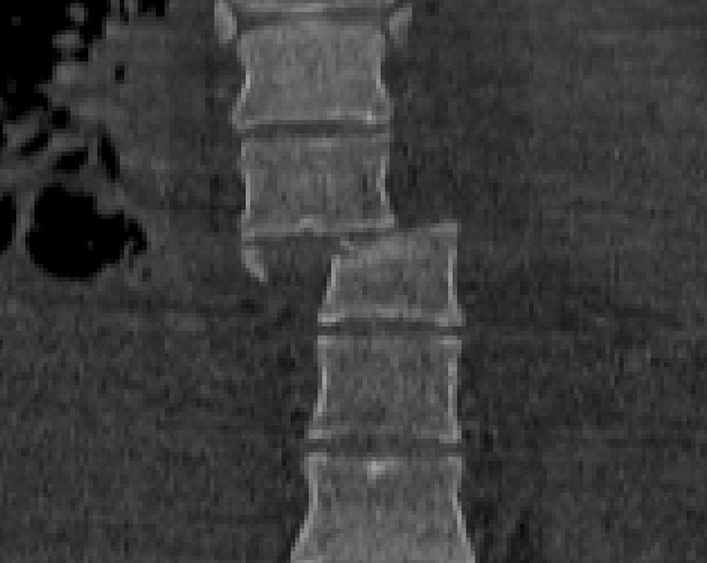
Rotational fracture-dislocation mechanism, CT scan, coronal view

**Fig. 5:**
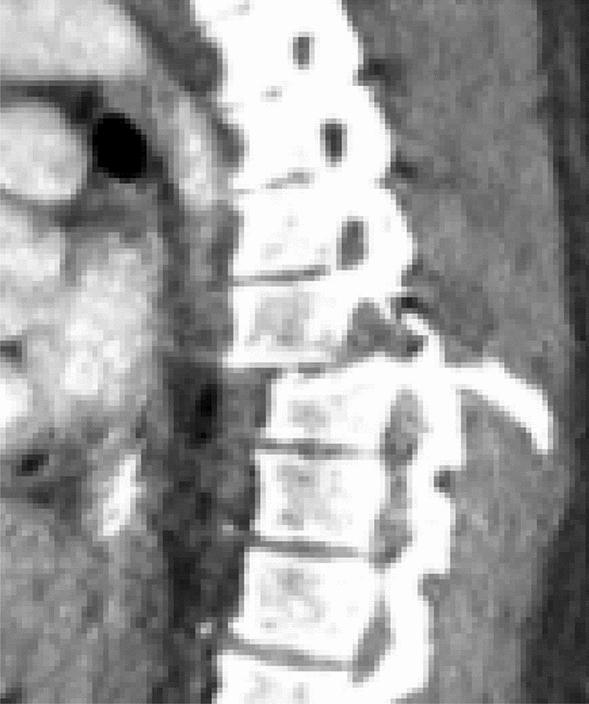
Rotational fracture-dislocation mechanism, CT scan, sagittal view

- *Rotational fracture dislocation mechanism* (Figs. 4, 5)

**Fig. 6:**
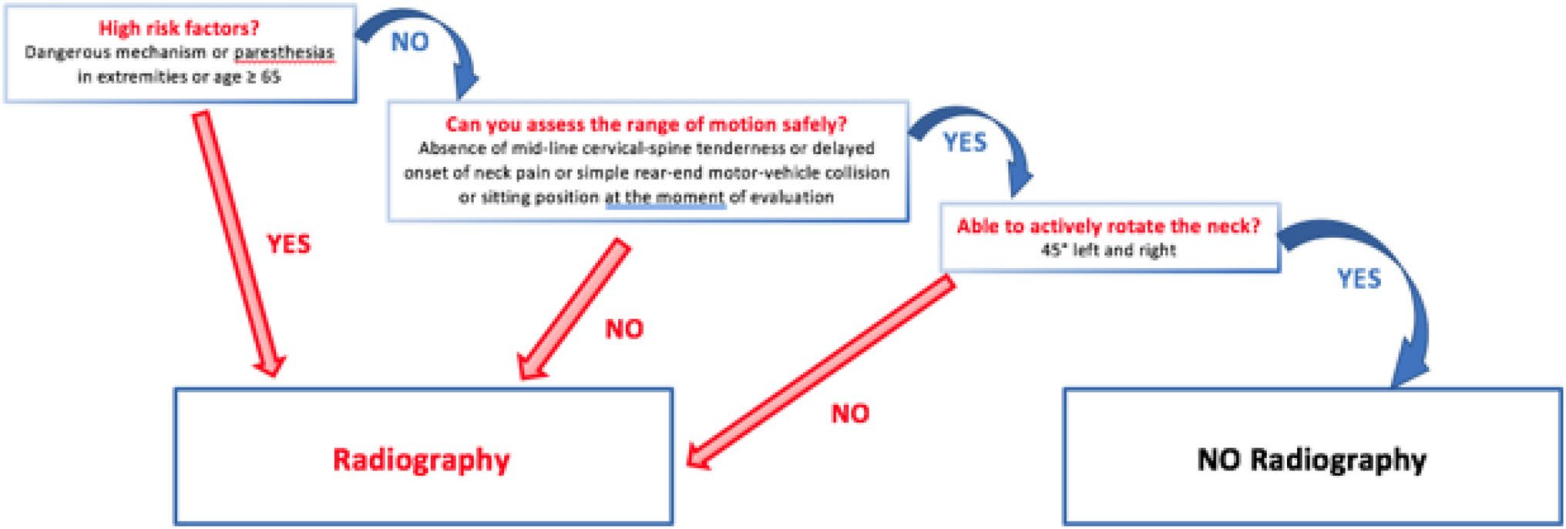
*The Canadian C-spine Rule*. For patients with a GCS (Glasgow Coma Scale) 15, hemodynamic stability, the presence of risk factors guides the role of imaging. Dangerous mechanisms describes falls ≥ 5 stairs, high-speed (> 100 km/h) impacts, ejections from vehicle, bicycle collision

This mechanism is an association of lateral flexion and rotation forces actin perpendicular to spinal axis, with often implied a component of posterior-anteriorly directed force. The injury pattern results in failure of both the posterior and middle columns with different degrees of anterior column damage. This causes the radiographic ‘‘slice’’ signs appearance [18, 20] (Fig. 6).

### Imaging modalities for cervical spinal trauma

In the emergency setting, the choice of adequate radiological imaging is secondary to the patient’s evaluation and depends on several factors. Among the most important are the patient’s clinical and neurological status including the careful assessment of pain, the presence of temporary or permanent neurological deficits, the type of trauma, and the patient’s own comorbidities.

#### Computer tomography (TC)

In this setting, CT is usually applied to study the whole spine and the CS [21]. Interesting and very useful is the possibility, especially in polytraumatized patients, to reconstruct the spine in its entirety and the cervical spine using the scans obtained to study the chest, abdomen, and pelvis with a sensitivity equivalent to that of the examination aimed at the spine. Compared to radiography, CT has a greater sensitivity in diagnosing fractures as well as being faster in terms of scans and technically easier to perform and undergo by a traumatized patient who should move as little as possible [22, 23].

#### Magnetic resonance imaging (MRI)

MRI, on the other hand, is the method of choice for the in-depth study of soft tissues, such as intervertebral ligaments and discs, vascular structures, and spinal cord [17, 24, 25].

Persistent neurological deficit after acute spinal trauma imposed a MRI to be performed, to exclude or confirm spinal cord injury or affection to other structures: such as hernias, paravertebral soft tissue edema, epidural hematomas. It is also an excellent test that can be used in the later stages of the therapeutic diagnostic process and to evaluate possible outcomes to get an idea of the prognosis.

### Role of imaging

The cervical spine has to be considered injured until proven otherwise in any polytrauma patient, and it is mandatory to rule out any spinal column injury before the safety removal of spinal precautions [22, 26, 27], either by clinical assessment or radiographic survey. A missed cervical spine injury may have potentially destructive consequences on patient outcome; therefore, the determination of cervical spine stability is mandatory in this setting [28].

The imaging of the spine is the mainly part of the initial management of acute spinal cord injury, but several issues of concern have to be relieved: who need imaging, what type of imaging [2].

Imaging of the cervical spine of every trauma patient results in radiation exposure and may be expensive considering its large use, whereas just few patients will have a spinal injury [26]. For this reason, criteria have been developed to decide whether the imaging is needed or not.

Thus, the National Emergency X-Ray Utilization Study (NEXUS) and the Canadian Cervical Spine study group suggested clinical criteria to select patients, which were adopted by the American College of Radiology in its appropriateness guidelines for screening patients before imaging the cervical spine [22].

According to a prospective cohort study performed in the Emergency Departments of nine Canadian tertiary care hospitals, the CCR is both more sensitive and more specific than the NLC when used for alert patients in stable condition. More specifically, the sensitivity of CCR was 99.4% while for NLC was 90.7%, the specificity for CCR was 45.1% and for NLC was 36.8% [29].

In elderly patients (over 65) and in patients with degenerative or anchylosing conditions, the biomechanical conditions of the spine may allow low-energy trauma to produce severe lesions. For this reason, in these patients we recommend to use a lower threshold for imaging use [22].

CS clearance on clinical grounds has converted into the standard of care in vigilant adult patients without midline cervical tenderness, neurologic symptoms, and distracting injuries. Indeed, regardless of the injury mechanism, awake and asymptomatic patients require no radiographic evaluation [26].

In addition, asymptomatic patient who is able to complete the physiologic range of motion may safely be cleared from cervical spine immobilization without imaging evaluation; this is because physical examination is a sensitive screening method for CS injury evaluation [30, 31].

The screening using Canadian C‑spine rule or NEXUS criteria has to be performed urgently in the setting of emergency department, and the images should be read by a skilled radiologist [32].

On the other hand, symptomatic trauma patients, complaining of neck pain, cervical spine tenderness, or showing neurological signs, and agitated or unconscious patients who cannot be assessed require radiographic study prior to the withdrawal of CS immobilization [26] (Table 2).

**Table 2:** Congress of Neurological Surgeons recommendations

Semeiotics	Imaging
*Awake and asymptomatic* not complaining of neck pain or tenderness, with a normal neurological examination and a complete functional range of motion at a physical examination	Radiographic evaluation of the cervical spine is not recommended and discontinuance of cervical immobilization is possible (Level I recommendation) [26]
*Awake and symptomatic*	High-quality computed tomography (CT) imaging is recommended If high-quality CT imaging is not available, 3 CS projections (anteroposterior, lateral, and odontoid) are recommended. Moreover, if necessary to define or better visualize suspicious areas, a CT is recommended, as soon as available (Level I recommendation) [26]
*Obtunded or unevaluable*	High-quality CT imaging is recommended as the initial imaging technique of choice If high-quality CT imaging is not available, 3 CS projections (anteroposterior, lateral, and odontoid) are recommended. Moreover, if necessary to define or better visualize suspicious areas, a CT is recommended, as soon as available (Level I recommendation) [26]

These assertions are further confirmed by the Congress of Neurological Surgeons recommendations [26].

As missed injury may have devastating consequences during last years, the imaging of choice had progressively shifted from conventional radiography to CT [20]. Indeed, multi-detector computer tomography (MDCT) has become the standard of care as initial screening to investigate blunt CS trauma patients who do not meet criteria for clinical clearance [33], and it is largely superior to all other imaging modalities to identify vertebral fractures and unstable anatomical conditions [34].

However, MR imaging is the gold standard to assess spinal cord and ligament injuries, as well as bone marrow edema [20].

The debate about the use of MRI in the acute setting of spinal cord injury remains: on the one side, it requires lot of resources to ensure 24 h availability, and most importantly, it requires more time for imaging acquisition, thus potentially delaying the surgery and putting at risk hemodynamically unstable patients. To minimize these risks, MRI studies are usually shortened in trauma setting [35].

Furthermore, MR angiography (MRA) can provide essential information about the presence of any vascular lesions, such as vertebral artery injury (VAI) or dissection, which can also modify initial management [35].

Anyway, in a prospective study, patients with a normal CT scan and no clinically significant injuries at the time of discharge did not seen modification of their management with the use of MRI [28]. That is to say that elimination of MRI from the routine workup in blunt trauma CS patient with normal CT scans who are awake, alert, and conscious is safe and cost-effective. Of course, if there is a neurological abnormality which could be attributable to spinal cord injury, MRI examinations after CT are required [32]. Therefore, more studies are needed to clear whether MRI role is beneficial or not in acute trauma setting [36].

#### Blunt cerebrovascular injuries (BCVI)

Up to 1% of non-penetrating neck traumas are complicated by blunt cerebrovascular injuries (BCVIs) [22]. BCVI can cause ischemic stroke with high mortality rates [37].

Patients can be asymptomatic at presentation and become symptomatic even at 72 h after trauma; others can be symptomatic at presentation despite an inconclusive diagnostic workup. Screening for these lesions with accurate imaging and clinical evaluation, with an early anticoagulation or antiplatelet therapy, can improve outcome and prevent the development of long-lasting deficit [22, 37].

CTA shows a level of accuracy sufficient to serve as an initial screening examination for blunt cerebro-vascular injuries [37] with a high sensitivity, which means that a negative CTA allows skilled radiologists to rule out significant artery injuries with a high degree of confidence. It is superior to conventional angiography, because it is faster, accurate and non-invasive so that in some trauma centers it is the screening modality of choice for suspected vascular injury [22].

A real concern, anyway, is the risk of false-positive results that can lead to an unnecessary anticoagulation or antiplatelet medications, so patients with questionable findings should undergo further evaluations [37]. Among them, even if screening criteria have changed over the time, mechanisms of injury are always included, such as high-speed deceleration accidents and consequent severe CS hyperextension/hyperflexion, or direct blunt trauma to head, face, neck, and upper chest [37].

However, there are findings that require further examinations, such as vertebral dislocations, severe hyperextension or hyperflexion, or fractures of CCJ or foramen trasversarium. In these cases, a screening by MDCTA may be required.

Concerning vertebral arteries injuries, the V2 segment is the most affected by dissection, compression, thrombosis, or pseudoaneurysm, with also a risk of distal embolization. Imaging findings could be an intimal flap, non-stenotic intraluminal irregularities, intraluminal occlusion, wall thickening by intramural hematoma, outpouchings of the arterial lumen (pseudoaneurysm) [22].

According to Biffi scale, which classifies the range of blunt cerebrovascular injuries based on CTA, there are 5 grades of injury:

- I–stenosis < 25% (dissection, intraluminal thrombus, intramural hematoma);
- II–stenosis > 25%;
- III–pseudoaneurysm;
- IV–occlusion
- V–vessel transaction or arterio-venous fistula

### Post-mortem studies

As in the clinical context, PMCT (post-mortem CT) is the most used technique when a cervical spine injury is suspected [38]. The advantage is mainly due to the very detailed appreciation of the upper CS bone lesions, thus improving the accuracy of the forensic investigation, guiding the subsequent autopsy [39]. In the same way, it allows studying any surgical treatment outcomes of this skeletal segment, often difficult to approach for the autopsy technique. Furthermore, the images can be produced, through a high-quality three-dimensional reconstruction, to be extremely illustrative, to present the reliefs in court.

These characteristics have allowed the PMCT to establish itself on the conventional radiological examination [40], previously used, characterized by less specificity and sensitivity so that some injuries, for example, cases with cranio-cervical dislocation and vertical laminar fractures, would have been omitted.

However, PMCT, as already extensively described in clinical settings, does not notice articular cartilage or the ligament complex, and intraspongious bone lesions may linger undiagnosed [39]. Again, PMCT is able to identify alterations in the skeletal segments, but it cannot provide certain information on the moment of production of the same (whether ante- or post-mortem) because the presence of blood in the muscles or connective tissues around the fracture is often seldom clearly noticeable [41].

Add to this the existence of nosological entities known as spinal cord injuries without radiographic abnormalities (SCIWORA), first described by Pang and Wilberger [42]. SCIWORA implied universally, albeit not unanimously, normal findings to techniques that resort to the use of ionizing radiation [43]. Therefore, MRI is universally recognized as the only imaging modality to detect this kind of cord injury.

Makino et al. [44] in their study identified five SCIWORA subjects who at autopsy had tiny hemorrhages affecting the spinal cord only microscopically detectable, without a complete transection or severe deformity, in the absence of other lethal lesions in all the other districts examined. Paraphrasing this evidence, it would therefore be said that the instrumental investigation was not in these cases able to identify the cause of death.

Currently, however, these structures (spinal cord, muscle tissue, cartilages, ligament structures, and soft tissues in general) could be better examined with MRI, although this has other limitations (non-standardized protocols, high cost, and time).

On the other hand, a review from Boese et al. [45] revealed a significant number of false negative at MRI examination of living subjects, so, analogously, false-negative MRI findings can also occur in postmortem cases.

To overcome these challenges, the study protocol of the corpse in cases of suspected cervical trauma should include a detailed and complete autopsy of histological and immunohistochemical investigations, preceded by CT or MRI.

Finally, from forensic pathology, a recent study [46] focused on the possibility of identifying new biomarkers of spinal trauma both for diagnostic purposes and as therapeutic targets: the mi-RNAs, whose behavior (up- or down-regulation), once standardized, could also allow the identification of false negative cases [47–52] as well as on the possible influences between these events and other systems and apparatuses of the organism [53–55].

However, further studies are needed to better understand the extent of this equipment, without forgetting that only through a multidisciplinary approach is it possible to reach diagnosis [56].

## Conclusions

The evolution, over time, of knowledge about spinal trauma and, specifically, cervical trauma, has made it possible to frame with greater precision the imaging techniques that can be used in the acute event.

It appears to be incontrovertible that CT and MRI play complementary roles in acute spinal trauma.

CT is the fastest, easiest, and most accessible first-line imaging modality. The information it provides regarding traumatic alterations of the normal anatomy, especially of the bone compartment, is fundamental for the clinician who has to decide the path of the traumatized patient.

For its part, MRI is unmatched in the evaluation of soft tissues such as discs, ligaments, and spinal cord. The use of magnetic resonance has well-defined indications in the acute trauma such as in the patient who has persistent neurological deficits or in the presence of persistence of pain in the case of inconclusive CT.

MRI has the extraordinary ability to reveal the location and severity of spinal cord injuries, especially in those patients who have incomplete injuries. In these cases, the precise finding of the lesion allows the further deterioration of the clinical condition.

The decision to use one or the other technique, when it comes to emergency urgency, must consider the stability or instability of the patient. MRI, while providing more information, will not be the first imaging choice in the unstable patient due to long imaging times compared to CT.

The advanced techniques provide greater and more detailed information about axonal and myelin integrity that are added to the information obtained from conventional sequences.

The evolution of imaging and neuroimaging techniques has made great strides in recent years.

However, if the imaging is negative and in front of a strongly indicative semeiotics, the existence of SCIWORA should not be forgotten [42–44].

In the next 50 years, this evolution promises further improvements, in part also borrowed from the evidence coming from forensic pathology, that will ensue an increasingly fine diagnostic accuracy and a better prognostic vision of the patient affected by spinal trauma.

## References

[1] Nuñez DB, Zuluaga A, Fuentes-Bernardo DA (1996). Cervical spine trauma: how much more do we learn by routinely using helical CT?. Radiographics.

[2] Como JJ, Diaz JJ, Dunham CM (2009). Practice management guidelines for identification of cervical spine injuries following trauma: update from the eastern association for the surgery of trauma practice management guidelines committee. J Trauma.

[3] Dreizin D, Letzing M, Sliker CW (2014). Multidetector CT of blunt cervical spine trauma in adults. Radiographics.

[4] Blackmore CC, Emerson SS, Mann FA, Koepsell TD (1999). Cervical spine imaging in patients with trauma: determination of fracture risk to optimize use. Radiology.

[5] Kwon BK, Vaccaro AR, Grauer JN (2006). Subaxial cervical spine trauma. J Am Acad Orthop Surg.

[6] Holdsworth F (1970). Fractures, dislocations, and fracture-dislocations of the spine. J Bone Joint Surg Am.

[7] Denis F (1983). The three column spine and its significance in the classification of acute thoracolumbar spinal injuries. Spine.

[8] Allen BL, Ferguson RL, Lehmann TR, O’Brien RP (1982). A mechanistic classification of closed, indirect fractures and dislocations of the lower cervical spine. Spine.

[9] Vaccaro AR, Hulbert RJ, Patel AA (2007). The subaxial cervical spine injury classification system: a novel approach to recognize the importance of morphology, neurology, and integrity of the disco-ligamentous complex. Spine.

[10] Patel AA, Dailey A, Brodke DS (2008). Subaxial cervical spine trauma classification: the Subaxial Injury classification system and case examples. Neurosurg Focus.

[11] Dvorak MF, Fisher CG, Fehlings MG (2007). The surgical approach to subaxial cervical spine injuries: an evidence-based algorithm based on the SLIC classification system. Spine.

[12] Joaquim AF, Lawrence B, Daubs M (2011). Evaluation of the subaxial injury classification system. J Craniovertebral Junction Spine.

[13] Patel AA, Hurlbert RJ, Bono CM (2010). Classification and surgical decision making in acute subaxial cervical spine trauma. Spine.

[14] Magerl F, Aebi M, Gertzbein SD (1994). A comprehensive classification of thoracic and lumbar injuries. Eur Spine J.

[15] Vaccaro AR, Koerner JD, Radcliff KE (2016). AOSpine subaxial cervical spine injury classification system. Eur Spine J.

[16] Wilmink JT (1999). MR imaging of the spine: trauma and degenerative disease. Eur Radiol.

[17] Daffner RH, Deeb ZL, Rothfus WE (1986). “Fingerprints” of vertebral trauma—a unifying concept based on mechanisms. Skelet Radiol.

[18] Parizel PM, Van Der Zijden T, Gaudino S (2010). Trauma of the spine and spinal cord: imaging strategies. Eur Spine J.

[19] Bensch FV, Koivikko MP, Kiuru MJ, Koskinen SK (2006). The incidence and distribution of burst fractures. Emerg Radiol.

[20] Izzo R, Popolizio T, Balzano RF (2019). Imaging of cervical spine traumas. Eur J Radiol.

[21] Tins B (2010). Technical aspects of CT imaging of the spine. Insights Imaging.

[22] ACR Appropriateness Criteria ^®^ on Suspected Spine Trauma - ClinicalKey. https://www.clinicalkey.com/#!/content/journal/1-s2.0-S1546144007004164. Accessed 9 Sep 2022

[23] Bernstein MP, Mirvis SE, Shanmuganathan K (2006). Chance-type fractures of the thoracolumbar spine: imaging analysis in 53 patients. AJR Am J Roentgenol.

[24] Flanders AE, Schaefer DM, Doan HT (1990). Acute cervical spine trauma: correlation of MR imaging findings with degree of neurologic deficit. Radiology.

[25] Sliker CW, Mirvis SE, Shanmuganathan K (2005). Assessing cervical spine stability in obtunded blunt trauma patients: review of medical literature. Radiology.

[26] Ryken TC, Hadley MN, Walters BC (2013). Radiographic assessment. Neurosurgery.

[27] Taghva A, Hoh DJ, Lauryssen CL (2012). Advances in the management of spinal cord and spinal column injuries. Spinal Cord Injury.

[28] Resnick S, Inaba K, Karamanos E (2014). Clinical relevance of magnetic resonance imaging in cervical spine clearance: a prospective study. JAMA Surg.

[29] Stiell IG, Clement CM, McKnight RD (2003). The Canadian C-spine rule versus the NEXUS low-risk criteria in patients with trauma. N Engl J Med.

[30] Anderson PA, Muchow RD, Munoz A (2010). Clearance of the asymptomatic cervical spine: a meta-analysis. J Orthop Trauma.

[31] Rose MK, Rosal LM, Gonzalez RP (2012). Clinical clearance of the cervical spine in patients with distracting injuries: it is time to dispel the myth. J Trauma Acute Care Surg.

[32] (UK) NCGC (2016) Spinal injury: assessment and initial management. Spinal Inj Assess Initial Manag

[33] Hoffman JR, Mower WR, Wolfson AB (2000). Validity of a set of clinical criteria to rule out injury to the cervical spine in patients with blunt trauma. National Emergency X-Radiography Utilization Study Group. N Engl J Med.

[34] Holmes JF, Akkinepalli R (2005). Computed tomography versus plain radiography to screen for cervical spine injury: a meta-analysis. J Trauma.

[35] Fehlings MG, Martin AR, Tetreault LA (2017). A clinical practice guideline for the management of patients with acute spinal Cord Injury: recommendations on the role of baseline magnetic resonance imaging in clinical decision making and outcome prediction. Glob spine J.

[36] Wu X, Malhotra A, Geng B (2018). Cost-effectiveness of magnetic resonance imaging in cervical clearance of obtunded Blunt Trauma after a normal computed tomographic finding. JAMA Surg.

[37] Anaya C, Munera F, Bloomer CW (2009). Screening multidetector computed tomography angiography in the evaluation on blunt neck injuries: an evidence-based approach. Semin Ultrasound CT MR.

[38] Cafarelli FP, Grilli G, Zizzo G (2018). Postmortem imaging: an update. Semin Ultrasound CT MR.

[39] Iwase H, Yamamoto S, Yajima D (2009). Can cervical spine injury be correctly diagnosed by postmortem computed tomography?. Leg Med (Tokyo).

[40] Stäbler A, Eck J, Penning R (2001). Cervical spine: postmortem assessment of accident injuries–comparison of radiographic, MR imaging, anatomic, and pathologic findings. Radiology.

[41] Bertozzi G, Maglietta F, Sessa F (2020). Traumatic brain injury: a forensic approach: a literature review. Curr Neuropharmacol.

[42] Pang D, Wilberger JE (1982). Spinal cord injury without radiographic abnormalities in children. J Neurosurg.

[43] Yucesoy K, Yuksel KZ (2008). SCIWORA in MRI era. Clin Neurol Neurosurg.

[44] Makino Y, Yokota H, Hayakawa M (2014). Spinal cord injuries with normal postmortem CT findings: a pitfall of virtual autopsy for detecting traumatic death. Am J Roentgenol.

[45] Boese CK, Nerlich M, Klein SM (2013). Early magnetic resonance imaging in spinal cord injury without radiological abnormality in adults: a retrospective study. J Trauma Acute Care Surg.

[46] Pinchi E, Frati A, Cantatore S (2019). Acute spinal cord injury: a systematic review investigating miRNA families involved. Int J Mol Sci.

[47] Sessa F, Maglietta F, Bertozzi G (2019). Human brain injury and miRNAs: an experimental study. Int J Mol Sci.

[48] De Matteis A, dell’Aquila M, Maiese A (2019). The Troponin-I fast skeletal muscle is reliable marker for the determination of vitality in the suicide hanging. Forensic Sci Int.

[49] Neri M, Fabbri M, D’Errico S (2019). Regulation of miRNAs as new tool for cutaneous vitality lesions demonstration in ligature marks in deaths by hanging. Sci Reports.

[50] Dell’aquila M, Maiese A, De Matteis A (2021). Traumatic brain injury: Estimate of the age of the injury based on neuroinflammation, endothelial activation markers and adhesion molecules. Histol Histopathol.

[51] La RR, Maiese A, Di Fazio N (2020). Post-Traumatic Meningitis Is a diagnostic challenging time: a systematic review focusing on clinical and pathological features. Int J Mol Sci.

[52] Pinchi E, Frati A, Cipolloni L (2018). Clinical-pathological study on β-APP, IL-1β, GFAP, NFL, Spectrin II, 8OHdG, TUNEL, miR-21, miR-16, miR-92 expressions to verify DAI-diagnosis, grade and prognosis. Sci Reports.

[53] Ferrara M, Bertozzi G, Zanza C (2022). Traumatic brain injury and gut brain axis: the disruption of an alliance. Rev Recent Clin Trials.

[54] Aromatario M, Torsello A, D’errico S (2021). Traumatic epidural and subdural hematoma: epidemiology, outcome, and dating. Medicina (Kaunas).

[55] Ferrara M, Bertozzi G, Volonnino G (2022). Glymphatic system a window on TBI pathophysiology: a systematic review. Int J Mol Sci.

[56] Ferrara M, Sessa F, Rendine M (2019). A multidisciplinary approach is mandatory to solve complex crimes: a case report. Egypt J Forensic Sci.

